# Study on the Genetic Diversity Characteristics of the Endemic Plant *Rhododendron bailiense* in Guizhou, China Based on SNP Molecular Markers

**DOI:** 10.1002/ece3.70966

**Published:** 2025-02-08

**Authors:** Jun Luo, Congjun Yuan, Haodong Wang, Jianhua Zhang, Jin Chen, Shuang He, Meng Chen, Xiaoyong Dai, Dali Luo

**Affiliations:** ^1^ Guizhou Academy of Forestry Guiyang Guizhou China; ^2^ College of Forestry Guizhou University Guiyang Guizhou China; ^3^ Guizhou Libo Karst Forest Ecosystem National Observation and Research Station Libo Guizhou China; ^4^ Panzhou Forestry Bureau Panzhou Guizhou China; ^5^ Key Laboratory of National Forestry and Grassland Administration on Biodiversity Conservation in Karst Mountainous Areas of Southwestern China Guiyang Guizhou China

**Keywords:** ddRAD‐seq, genetic structure, population dynamic history, single nucleotide polymorphism

## Abstract

*Rhododendron bailiense* was identified as a new species in 2013, with approximately 150 individuals existing globally, found only in Dafang County and Panzhou City, Guizhou Province, China. Despite its discovery, the genetic diversity and population structure of this species remain poorly understood, hindering efforts to collect and conserve wild germplasm resources. In this study, double digest restriction‐site associated DNA sequencing was conducted on 26 samples from two populations of *R. bailiense* to identify single nucleotide polymorphism (SNP) loci. Using these data, the research explores the genetic diversity and structure of *R. bailiense* populations and infers their population dynamics and evolutionary history. The results indicate that *R. bailiense* has a moderate level of genetic diversity (*π* = 0.2489, *H*_*o*_ = 0.2039, *H*_*e*_ = 0.2331). Genetic differentiation between populations is relatively high (55.94%), with a genetic differentiation coefficient (*F*_*ST*_) of 0.1907. This suggests that *R. bailiense* historically might have been a large population, which, due to geological historical events, became fragmented into the two existing populations. The Panzhou population demonstrates a heterozygote selection advantage. Conversely, the Dafang population faces the risk of inbreeding depression, further exacerbated by its limited gene flow. Consequently, in situ conservation is recommended for the Panzhou population, while ex‐situ conservation is suggested for the Dafang population. Additionally, research on breeding techniques is necessary to expand the population size while maintaining high genetic diversity.

## Graphical abstract

This study is the first to use ddRAD‐seq sequencing technology for Single Nucleotide Polymorphism (SNP) analysis, systematically analyzing the genetic background of R. bailiense. This species exhibits rich genetic diversity at both species and population levels. This study provides a theoretical basis for the conservation of wild resources of the R. bailiense and lay the foundation for the breeding or cultivation of this new species.

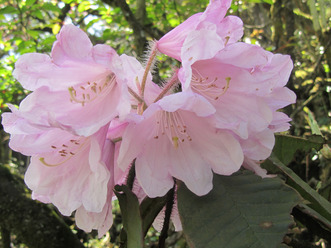

## 1 Introduction

**FIGURE 1:**
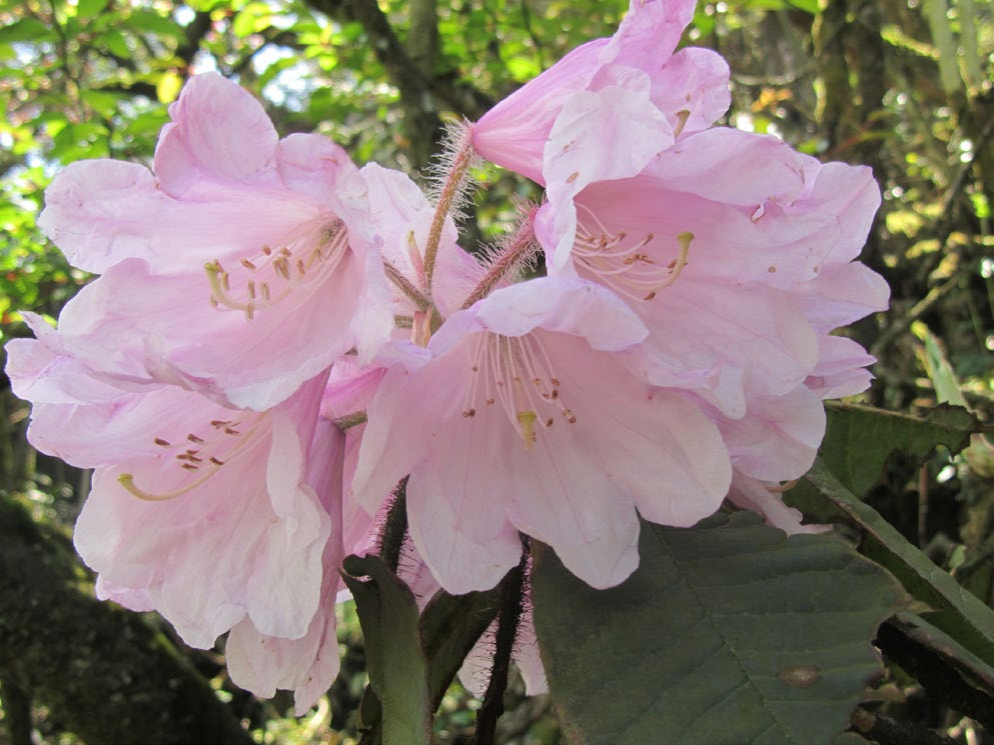
A photograph of *R. bailiense*.

*Rhododendron bailiense* Y.P.Ma, C.Q.Zhang, and D.F.Chamb. was a new species first discovered in 2013 by the Kunming Institute of Botany, Chinese Academy of Sciences, and the Royal Botanic Garden Edinburgh (Figure 1) (Ma, David, et al. 2015). This species belongs to the Auriculata subsection of the Rhododendron genus and grows only in slightly alkaline soils at elevations between 1800 and 2080 m (Pang et al. 1993; Fang and Min 1995; Yang et al. 2006; Zhang et al. 2024). According to our survey, this species has a narrow distribution, found only in Dafang County and Panzhou City in Guizhou Province, with fewer than 50 mature individuals in the wild, exclusively growing on limestone mountains in karst areas (Yang et al. 2020; Dai et al. 2022). The survival status of this species is concerning, and urgent conservation efforts are needed. To this end, the Guizhou Provincial Wildlife Conservation Association is actively advocating to the National Forestry and Grassland Administration and the People's Government of Guizhou Province to include it in the list of key protected species and to increase protection efforts for this species.

The magnitude of genetic diversity is the result of long‐term biological evolution and is a prerequisite for the survival, development, and evolution of organisms (BGCI 2021; Sun et al. 2023; Pereira et al. 2024). Conservation of genetic resources and plant breeding programs required assessments of the genetic diversity and structure of species (Primack and Ma 2009; He et al. 2024). However, past studies only reported on the community characteristics of *R. bailiense*, and there were no reports on research focusing on population structure and genetic diversity. Due to the lack of genetic background knowledge, it was difficult to plan conservation strategies for this plant (Sokal et al. 1989; Wuyun et al. 2015; Ma et al. 2019; Sun et al. 2020).

The use of molecular markers can accurately estimate genetic diversity (Tong et al. 2020; Liu et al. 2021). In the past, researchers have utilized various molecular markers, including Simple Sequence Repeats (SSR), Random Amplified Polymorphic DNA (RAPD), Amplified Fragment Length Polymorphism (AFLP), Inter‐Simple Sequence Repeats (ISSR), and Restriction Fragment Length Polymorphism (RFLP) to analyze the genetic diversity of plants (Powell et al. 1996; Zhang et al. 2019; Garcia‐Mas et al. 2000; Garcia et al. 2004; Shi et al. 2023; Tang et al. 2023). Among the various molecular marker techniques, Single Nucleotide Polymorphism (SNP) markers are widely used due to their numerous polymorphic sites, broad distribution, strong genetic stability, and high sequencing throughput (Deschamps et al. 2012; Helyar et al. 2015; Zhou et al. 2018). These advantages have led to their extensive application in the genetic diversity analysis of species such as *Pinus bungeana* (Tian et al. 2021), *Humulus lupulus* (Zhao et al. 2021), *Zea mays* (Xue et al. 2021), *Glycine max* (Yang et al. 2021), *Primula sikkimensis* (Chen 2021), *Oryza sativa* (Xu 2016), and *Capsicum annuum* (Liu et al. 2014). Moreover, SNP markers are also employed in population genetics research for Rhododendron species such as *R. cyanocarpum* (Liu et al. 2020), *R. sinofalconeri* (Zhang et al. 2021), and *R. hemsleyanum* (Cao et al. 2022). Additionally, the double digest restriction‐site associated DNA sequencing (ddRAD‐seq) technique is commonly used for SNP site development due to its simplicity of operation, quick library construction, high throughput, and low experimental costs, providing sufficient informative sites for genetic background studies (Hosoya et al. 2018; Lavretsky et al. 2019; Sudan et al. 2019; Janjua et al. 2020; Liu et al. 2022).

This study utilized double digest restriction‐site associated DNA sequencing (ddRAD‐seq) technology to perform Single Nucleotide Polymorphism (SNP) molecular marker analysis on the genetic diversity indices, genetic differentiation, population structure, and historical dynamics of the existing two *R. bailiense* populations. Our results aimed: (1) to systematically reveal the genetic diversity and historical population dynamics of *R. bailiense*; (2) to analyze the causes of the current genetic structure of the species; (3) to provide an appropriate conservation and management strategy.

## 2 Materials and Methods

### 2.1 Plant Materials

**FIGURE 2:**
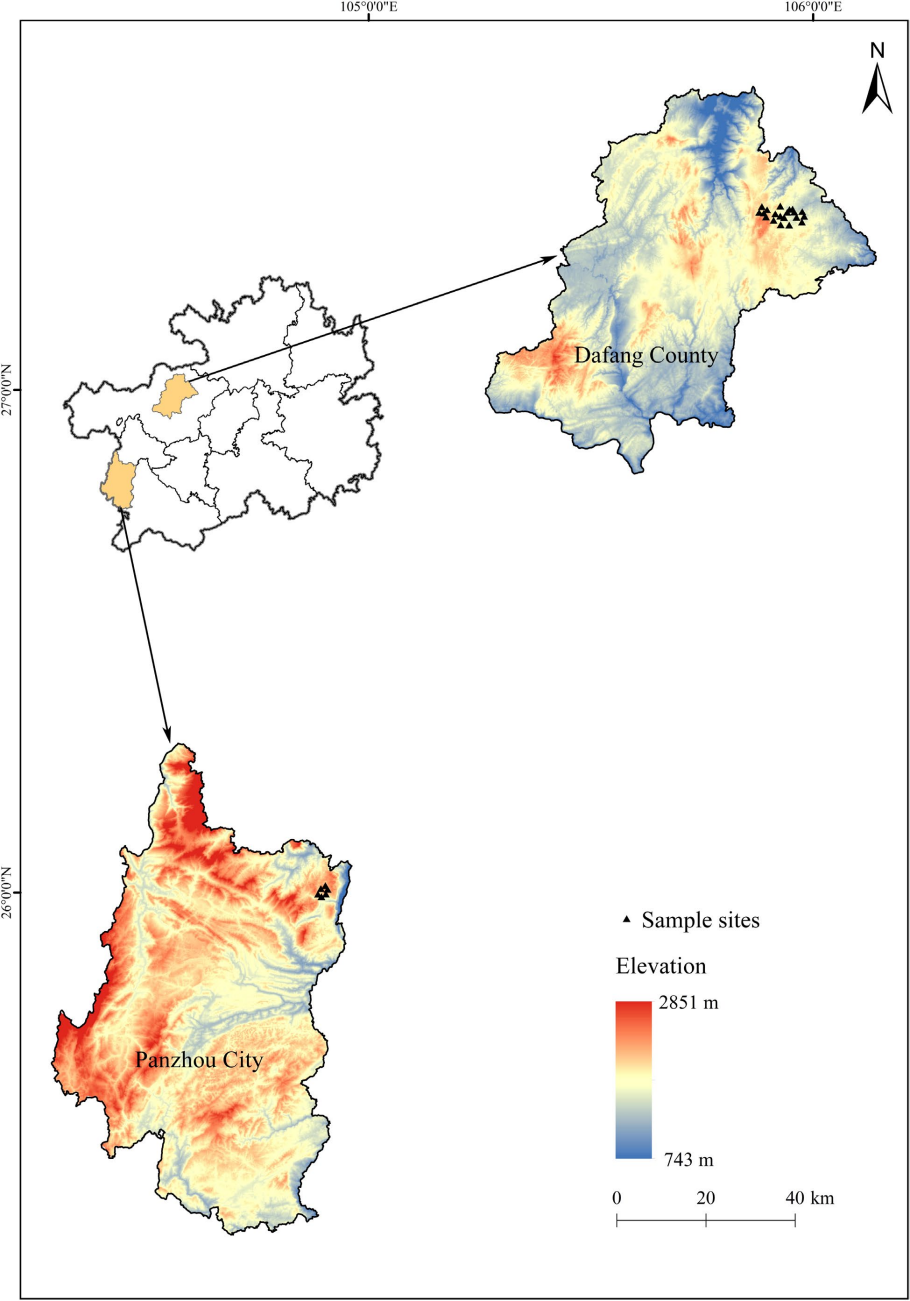
Geographic locations of *R. bailiense* populations sampled in this study.

The 26 *R. bailiense* materials used in this study were collected from two different populations in Guizhou province, with 20 samples from Dafang population (DF) and 6 from Panzhou population (PZ). Details of the sampling are provided in Table 1 and Figure 2. Sample collection followed the principles and methods of population genetics, adhering to the principles of representativeness and comparability (at least 5 m apart) (Wang et al. 2019). Fresh leaves were collected, labeled with sample numbers, then placed in sealed storage bags and stored in a cooling box for transport back to the laboratory. Subsequently, these samples were rapidly frozen using liquid nitrogen and preserved in a freezer at −80°C to extract DNA. The formal identification of the samples used in this study was performed by Dai Xiao‐Yong. Voucher specimens were deposited in the Herbarium of Guizhou Provincial Academy of Forestry (GF) and Kunming Institute of Botany, Chinese Academy of Sciences (PE), the deposition number was 1,550,524 (fl., Holotype GF!, isotypes KUN!, PE!). Our field investigation and experimental studies comply with the regulations of local legislative bodies, national and international guidelines.

**TABLE 1:** The sampling information of six populations of *R. bailiense*.

Population	Sample locality	City (state)	Longitude (E)	Latitude (N)	Altitude/m	AMT/°C	AMP/mm	Sample size
DF	Dafang county	Bijie	105°52′	27°17′	1780	11.8	1155	20
PZ	Panzhou city	Liupanshui	104°55′	26°02′	2040	15.2	1390	6

### 2.2 DNA Extraction and ddRAD Library Construction

Total genomic DNA was extracted using the cetyl trimethyl ammonium bromide (CTAB) method (Doyle and Doyle 1990). DNA integrity was checked by 0.8% agarose gel electrophoresis, and DNA concentration and purity were measured using the Promega QuantiFluor fluorescence quantimeter.

The ddRAD library was constructed following the method described by Peterson et al. (2012). DNA was diluted to 10 ng/μL and digested with EcoRI and MseI enzymes at 37.5°C for 5 h, followed by a temperature increase to 65°C for 20 min in a PCR machine to inactivate the enzymes. After the temperature was reduced to 12°C, T4 ligase and adapters (NEB) were added, and ligation was performed at 16°C for 4 h, followed by enzyme inactivation at 65°C for 20 min, and then held at 12°C. The ligation products were mixed in equal volumes and separated in a 2% agarose gel. DNA fragments ranging from 220 to 450 bp were excised and recovered. The gel was dissolved in a water bath and fragments were recovered using a gel extraction kit (Omega Inc., USA). The purified products were PCR amplified to the desired concentration and sequenced on an Illumina NovaSeq with 150 bp paired‐end sequencing (generating approximately 0.5 Gb of data per sample). DNA extraction and ddRAD‐seq library preparation were performed by Personal Biotech Co. Ltd. (Shanghai, China).

### 2.3 SNP Locus Mining

ddRAD sequencing data were processed using Stacks v.2.0 (Falush et al. 2003; Catchen et al. 2013; OLeary et al. 2018; Rochette et al. 2019). Initially, the process_radtags module was utilized for data quality control, removing low‐quality sequences and sequences without rad tags from the raw data. The ustacks module was then used to cluster and deduplicate individual sample data, retaining similar sequences that met the required sequencing depth and mismatch conditions to form loci (parameters set as follows: ‐min depth of coverage required to create a stack (m): 4−max distance allowed between stacks (*M*): 2−max distance allowed to align secondary reads to primary stacks: 4−max number of stacks allowed per de novo locus: 3−minimum alignment length: 0.8; −model type: SNP; −alpha significance level for model: 0.01). The cstacks module was used to merge loci obtained from all individuals into catalogs with default parameters. The sstacks module was then run to align individual sample loci to catalog loci, generating SNP, allele, and tags information for each sample. SNP calling was performed using the populations module (parameters set as follows: ‐min number of populations a locus must be present in to process a locus (*r*): 8−min percentage of individuals in a population required to process a locus for that population (*p*): 0.75; −max observed heterozygosity required to process a nucleotide site at a locus: 0.6−min minor allele frequency required to process a nucleotide site at a locus: 0.02), thereby filtering out SNP loci.

### 2.4 Data Analyses

Tajima's D values were calculated using vcftools v.0.1.15 with a 95% confidence interval (−1.082 to 2.039) to perform neutrality tests on the obtained SNPs, setting the sliding window size to 3000 bp (Fu 1997).

Genetic diversity parameters, including observed heterozygosity (*H*_*o*_), expected heterozygosity (*H*_*e*_), nucleotide diversity (*π*), and inbreeding coefficient (*F*_*is*_), were calculated using the populations module of the Stacks software (Catchen et al. 2013). Molecular variance analysis (AMOVA) of SNPs was conducted using the Arlequin v.3.5.2 software to estimate genetic differentiation coefficients (*F*_*ST*_) between populations for population genetic diversity analysis (Excoffier and Lischer 2010).

The genetic structure of populations was analyzed using admixture software (http://dalexander.github.io/admixture) based on SNP data (Alexander et al. 2009; Du et al. 2024), setting *K* = 2 ~ 20 (assuming 2 ~ 20 ancestral populations) and choosing a mixed model with other parameters set to default. The CV error values for different Ks were used to determine the *K* closest to the real value for population genetic structure analysis.

Pairwise identical by state (IBS) similarity between individuals was calculated using plink (v1.9), generating an IBS similarity matrix and subsequently a genetic distance matrix (=1‐IBS), from which a Kinship heatmap was drawn (Geng et al. 2021). The Genomic relationship matrix values between pairs of individuals were calculated using Gmatrix (Ver2) software, and a Kinship heatmap was drawn to analyze kinship relationships (Martin et al. 2008).

Principal Component Analysis (PCA) based on SNP data (excluding SNPs with MAF less than 0.05) was performed using GCTA software (http://www.complextraitgenomics.com/software/gcta/) (Chang et al. 2015). A phylogenetic tree was constructed using the Maximum likelihood algorithm in FastTree software (http://www.microbesonline.org/fasttree/) (Steel and Rodrigo 2008), followed by cluster analysis. After tree construction, the reliability of the phylogenetic tree branches was validated (bootstrap, 1000 replications).

The script easySFS (https://github.com/isaacovercast/easySFS) was used to convert VCF to SNP frequency spectrum (SFS) format. Stairway Plot v.2.1.2 was used to infer changes in effective population size (*N*_*e*_) over time using 67% of randomly selected loci from all sites (Liu and Fu 2015; Nielsen 2000). Previous research indicates that the generation time for Rhododendron species is generally about 10 years, such as for *R. ponticum* (Cross 1975), which requires approximately 10 years from seedling to fruiting. Liu et al. (2020) found similar statistical models when simulating the population dynamic history of *R. cyanocarpum* with generation times set at 10, 20, and 30 years. According to research by Yoichi et al. (2016), the mutation rate for *R. weyrichii* is 1.581 × 10^−9^. Thus, the generation time for *R. bailiense* was set at 10 years and the mutation rate at 1.581 × 10^−9^.

## 3 Results

### 3.1 ddRAD Sequencing Analysis

Through ddRAD sequencing, a total of 13.1 Gb of sequencing data were obtained from 26 *R. bailiense* samples. After filtering with fastp (v0.20.0) to remove low‐quality sequences and reads without tags, a total of 61.0 Mb of high‐quality reads data was retained. As Table 2 shows, the average proportion of high‐quality reads to original reads per sample is 96.19%, and the average proportion of high‐quality bases to original bases is 94.97%. This indicates that the paired‐end sequencing sequences can be fully mapped to the reference genome, confirming the success of the ddRAD library construction. The average GC content of the sequencing is 42.83%, with an average base quality of Q20 at 97.54%, and Q30 at 93.04%. The average sequencing depth is 51.67x, indicating a low error rate and high data quality.

**TABLE 2:** *R. bailiense* ddRAD sequencing data.

Sample	Reads number	HQ reads number (%)	Base number	HQ base number (%)	Sequencing depth (*x*)	GC (%)	Q20 (%)	Q30 (%)
df2	15,504,770	97.35	2,201,799,840	96.33	57.5	41.98	97.88	93.66
df3	17,878,186	97.18	2,539,968,144	96.14	60.24	42.23	97.78	93.5
df6	8,023,564	95.32	1,123,298,960	94.02	54.68	43.39	97.36	92.83
df10	7,772,044	95.4	1,088,086,160	94.07	53.09	43.21	97.36	92.83
df11	7,433,438	95.78	1,040,681,320	94.51	49.78	43.47	97.44	92.87
df12	8,298,610	95.79	1,161,805,400	94.44	56.22	43.21	97.31	92.57
df13	8,566,166	96.16	1,199,263,240	94.89	53.2	42.79	97.51	92.99
df14	7,610,972	96.3	1,065,536,080	95.09	50.21	42.85	97.62	93.25
df15	8,079,004	95.48	1,131,060,560	94.14	52.99	43.26	97.32	92.67
df16	12,709,990	95.96	1,800,120,938	94.77	49.53	40.88	97.43	92.68
df17	16,446,018	96.71	2,332,726,529	95.56	60.84	40.38	97.61	93.1
df18	7,741,538	95.86	1,083,815,320	94.63	51.42	42.84	97.51	93.04
df19	7,812,032	95.8	1,093,684,480	94.56	46.42	43.3	97.5	93.04
df20	7,378,918	97.74	1,033,048,520	96.75	47.66	42.44	97.87	93.46
df21	7,559,506	97.67	1,058,330,840	96.65	48.66	42.44	97.85	93.48
df22	7,912,520	95.94	1,107,752,800	94.77	46.88	43.16	97.54	93.05
df23	7,640,318	95.87	1,069,644,520	94.63	49.76	43.22	97.4	92.65
df24	7,258,976	95.64	1,016,256,640	94.42	46.72	43.14	97.44	92.91
df25	7,757,786	96.2	1,086,090,040	94.91	52.35	42.49	97.6	93.27
df26	8,212,512	95.72	1,149,751,680	94.44	49.55	43.07	97.31	92.48
pz1	8,415,188	95.97	1,178,126,320	94.61	52.34	43.37	97.41	92.8
pz3	8,058,186	95.76	1,128,146,040	94.38	50.08	43.79	97.31	92.55
pz4	7,554,840	96.02	1,057,677,600	94.79	53.44	43.41	97.6	93.34
pz5	8,800,764	96.25	1,232,106,960	94.97	54.73	42.98	97.55	93.08
pz6	8,751,128	96.86	1,225,157,920	95.67	51.78	42.67	97.85	93.7
pz8	7,450,808	96.27	1,043,113,120	95.06	43.32	43.58	97.65	93.34
Average	9,101,069	96.19	1,278,732,691	94.97	51.67	42.83	97.54	93.04

*Note:* GC: The proportion of cytosine and guanine in the genome; Q20: Percentage of bases with an accuracy of more than 99% for base recognition; Q30: Percentage of bases with an accuracy of more than 99.9% for base recognition.

### 3.2 SNP Analysis

Using the ustacks module, clustering and deduplication were performed on the sample data, resulting in a total of 3,993,634 loci. The cstacks module was used to merge the loci obtained from all individuals with default settings, yielding 1,664,624 catalog loci. The populations module was run to filter for common SNP sites, obtaining a total of 813,714 SNP sites. As shown in Table 3, there were 258,528 SNP sites that underwent transitions between pyrimidines or between purines, with a transition rate of 31.77%. There were 133,380 SNP sites where transversions occurred between a purine and a pyrimidine, with a transversion rate of 16.39%. Transitions greatly outnumbered transversions, with an average transition‐to‐transversion ratio (Ts/Tv) of 1.94, indicating that SNP mutations are mainly influenced by purifying selection and transitions are more common than transversions.

**TABLE 3:** Transition and transversion statistics of SNPs.

Sample	SNP number	Ts	Tv	Ts/Tv
df2	31,112	9177	4943	1.86
df3	31,367	9349	4794	1.95
df6	30,072	9763	4928	1.98
df10	31,249	9900	5081	1.95
df11	31,380	9615	5026	1.91
df12	31,754	9452	4967	1.9
df13	31,743	9837	5192	1.89
df14	30,386	9145	4778	1.91
df15	31,145	9946	5177	1.92
df16	31,119	9496	4937	1.92
df17	32,212	9682	5003	1.94
df18	32,086	9819	5085	1.93
df19	31,186	10,203	5134	1.99
df20	30,374	9039	4780	1.89
df21	30,570	8921	4709	1.89
df22	31,809	10,150	5275	1.92
df23	31,638	9674	5148	1.88
df24	31,848	9407	4984	1.89
df25	31,910	9311	4903	1.9
df26	32,221	9707	4998	1.94
pz1	31,541	11,753	5923	1.98
pz3	30,989	11,162	5579	2
pz4	31,272	9879	4867	2.03
pz5	31,182	11,308	5650	2
pz6	31,116	11,287	5741	1.97
pz8	30,433	11,546	5778	2
Average	31,297	9943	5130	1.94
Total	813,714	258,528	133,380	50.34

Abbreviations: Ts, transitions between nucleotides of the same kind; Tv, transversions between different nucleotides.

**FIGURE 3:**
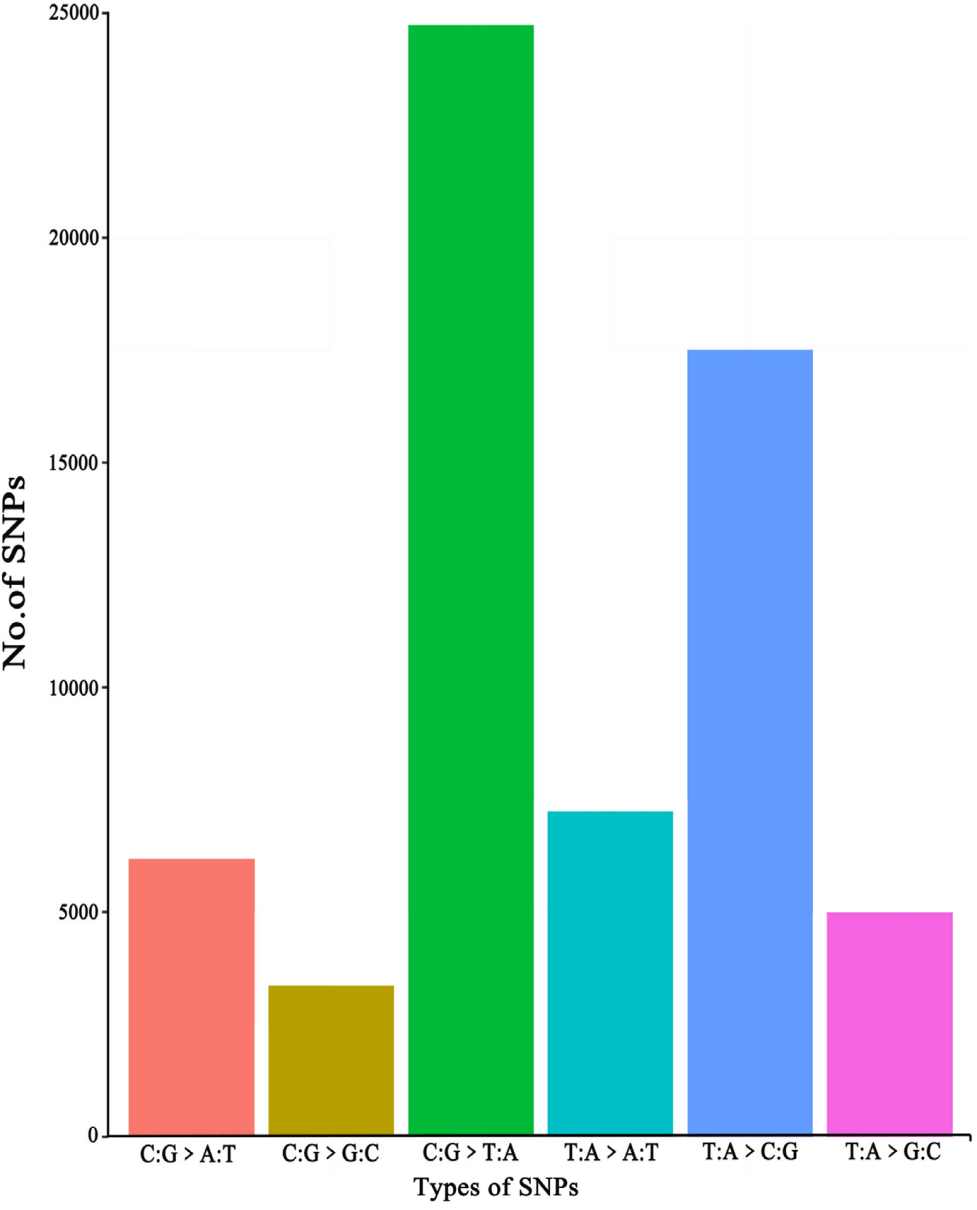
SNP mutation spectrum analysis.

Through mutation spectrum analysis (Figure 3), genomic SNP mutations can be classified into six categories, with T: A>C: G and C: G>T: A being the primary SNP mutation types. Tajima's D test was performed on the obtained SNPs (Table 4), with an average Tajima's D value of −1.4238 at the species level based on all sites, and the P‐value was significant (*p* < 0.05), suggesting a high frequency of rare alleles (Tajima's D < 0) (Krutovsky and Neale 2005). This indicates that the genetic diversity within the *R. bailiense* population is lower than expected under neutral evolution, possibly due to past population expansions.

**TABLE 4:** The results of Tajima's *D* tests.

Population	Tajima's *D*	*p*‐value
DF	−1.2545	0.073
PZ	−1.5931	0.000
Average	−1.4238	0.036

### 3.3 *R. Bailiense* Population Genetic Diversity Analysis

Table 5 lists the genetic diversity parameters for two populations of *R. bailiense*. The average values for nucleotide diversity (*π*), observed heterozygosity (*H*_*o*_), and expected heterozygosity (*H*_*e*_) for the populations studied are 0.2489, 0.2039, and 0.2331, respectively. In the Dafang population (DF), the nucleotide diversity (*π*) value is 0.2907, with observed heterozygosity (*H*_*o*_) less than expected heterozygosity (*H*_*e*_), and an inbreeding coefficient (*F*_*is*_) of 0.2184, indicating that the DF population has high genetic diversity, and some degree of inbreeding or close breeding. For the Panzhou population (PZ), the nucleotide diversity (*π*) value is 0.2070, with observed heterozygosity (*H*_*o*_) greater than expected heterozygosity (*H*_*e*_), and an inbreeding coefficient (*F*_*is*_) of 0.0395, suggesting that the PZ population has lower genetic diversity and a heterozygote advantage.

**TABLE 5:** Population genetic statistics of *R. bailiense*.

Population	*H*_*o*_	*H*_*e*_	*π*	*F*_*is*_
DF	0.2173	0.2815	0.2907	0.2184
PZ	0.1905	0.1847	0.2070	0.0395
Average	0.2039	0.2331	0.2489	0.1290

Abbreviations: *π*, Nucleotide diversity; *F*_*is*_, Inbreeding coefficient within population; *H*_*e*_, Expected heterozygosity; *H*_*o*_, Observed heterozygosity.

### 3.4 Genetic Differentiation

The calculated genetic differentiation coefficient (*F*_*ST*_) between populations of *R. bailiense* is 0.1907, indicating significant genetic differentiation between populations (0.15 < *F*_*ST*_ < 0.25). This finding is also supported by the analysis of molecular variance (Table 6), with 55.94% of the genetic variation originating among populations and 44.06% originating within populations. This suggests that there is a high degree of differentiation between *R. bailiense* populations, with the main source of genetic variation arising from between populations.

**TABLE 6:** Analysis of molecular variance (AMOVA) in the wild populations of *R. bailiense*.

Source	df	Sum of squares	Variance components	Percentage of variation (%)
Among populations	1	21694.22	2165.41	55.94
Within populations	24	40938.93	1705.79	44.06
Total	25	62633.15	3871.20	

### 3.5 Analysis of the Genetic Structure of *R. Bailiense* Populations

**FIGURE 4:**
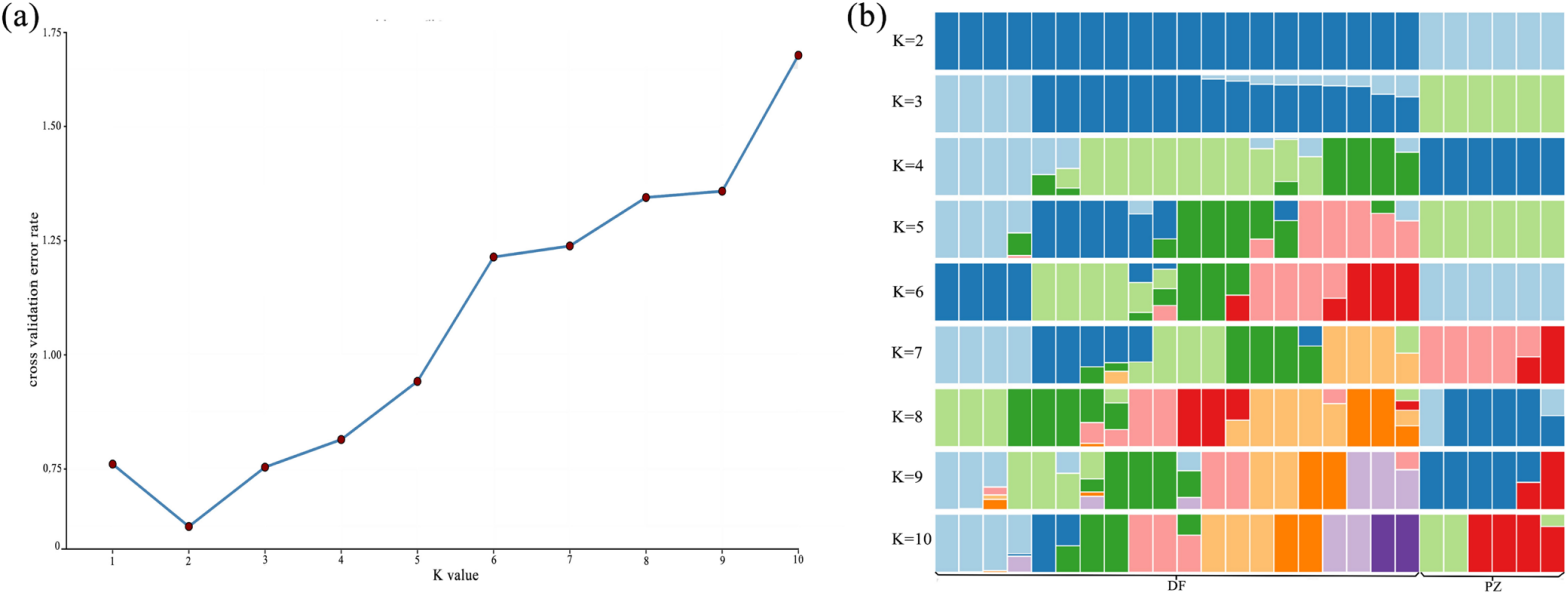
(a) Distribution of CV error values corresponding to different K values. (b) Diagram of the genetic structure of the *R. bailiense* macrophyllum population.

This study analyzed the population genetic structure using the Admixture software to tag SNP data, followed by Bayesian forecasting model for population structure analysis (Figure 4). When cross‐validation of clustering at *K* = 2, all materials were divided into two subgroups: the first subgroup consisted of 20 samples from Dafang County, and the second subgroup included 6 samples from Panzhou City. When cross‐validation of clustering at *K* = 5, all materials were divided into five subgroups, with four subgroups originating from Dafang and the fifth subgroup still from Panzhou City. At *K* = 10, all materials were divided into 10 subgroups, where the genetic genes and genetic compositions between subgroups became more complex. The closer the maximum likelihood value is to zero, the more accurate the *K* value is in reflecting the actual situation. In this study, the maximum likelihood value was closest to zero at *K* = 2, suggesting that the *R. bailiense* population has two genetic structures, thus supporting the hypothesis that the DF and PZ populations may have originated from two different ancestors.

### 3.6 Kinship Analysis

**FIGURE 5:**
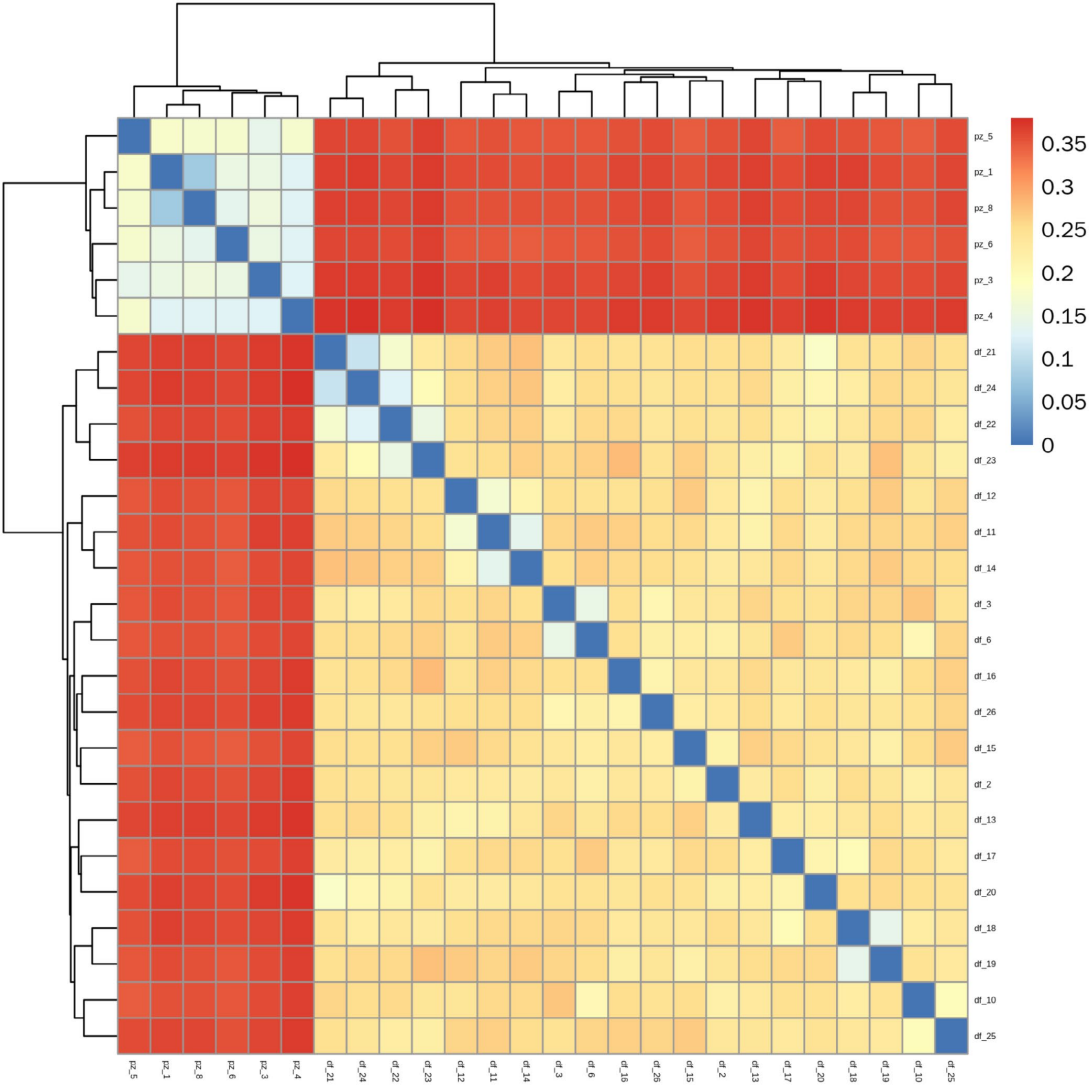
*R. bailiense* Population 1‐IBS genetic distance matrix heatmap.

This study used plink (v1.9) to calculate the IBS (Identity by State) similarity between pairs of individuals, generating a 1‐IBS genetic distance matrix. Each small square represents the genetic distance between two samples; the closer the color to blue, the smaller the distance and the closer the relationship; the redder the color, the greater the distance and the more distant the relationship. As shown in Figure 5, the PZ population exhibits smaller genetic distances and closer kinship among its samples. Conversely, the DF population shows larger genetic distances and more distant relationships between its samples, with a clear heterosis advantage.

**FIGURE 6:**
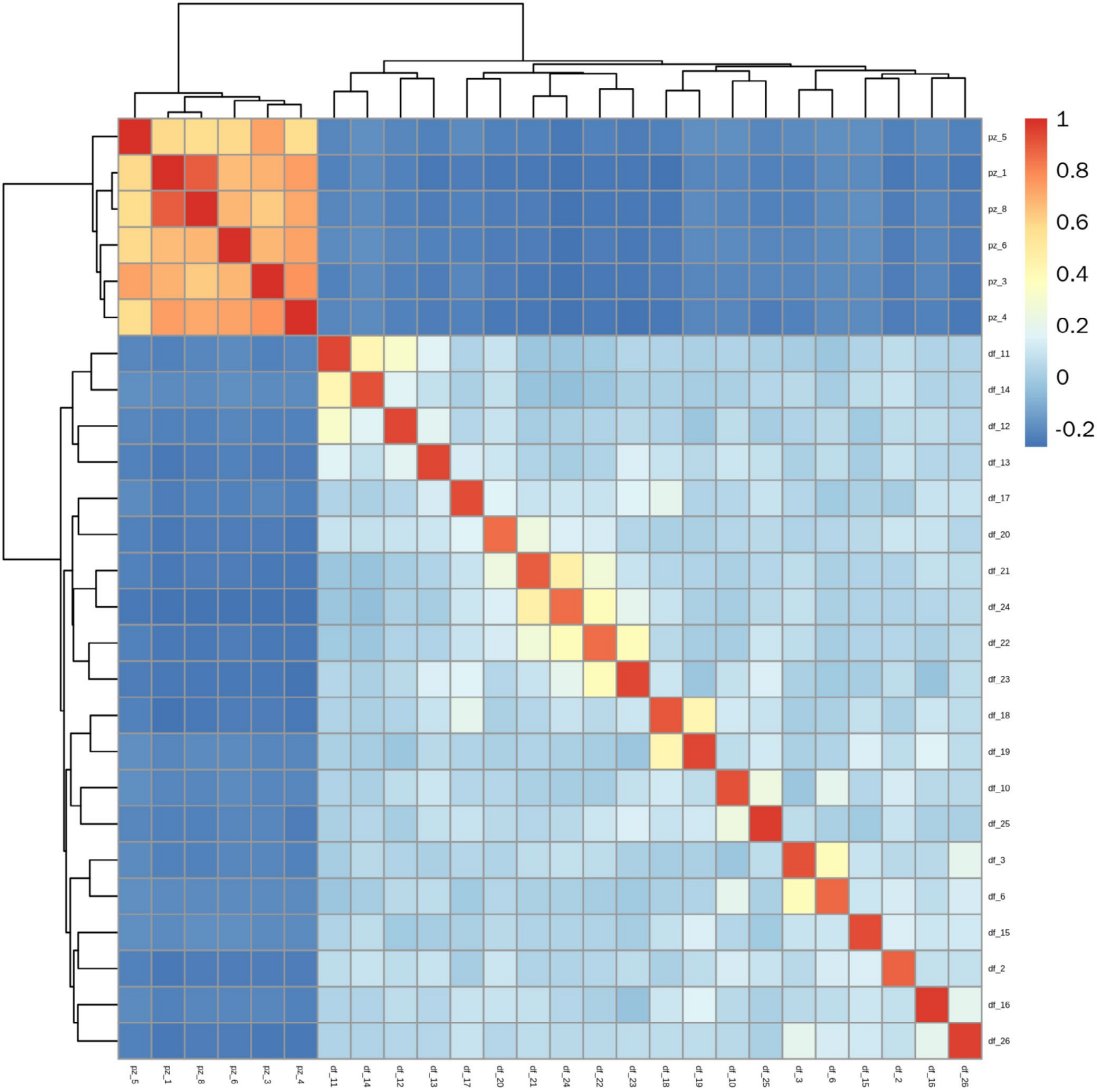
*R. bailiense* Population G‐value matrix heatmap.

Using Gmatrix (Ver2) to calculate the G values between pairs of individuals, a whole‐genome relationship matrix was generated. Each small square represents the G value between two samples; the closer the color to red, the larger the G value and the closer the kinship; the bluer the color, the smaller the G value and the more distant the kinship. As seen in Figure 6, the PZ population has larger G values and closer relationships among its samples. In contrast, the DF population's samples have smaller G values and more distant relationships.

### 3.7 Principal Component Analysis

**FIGURE 7:**
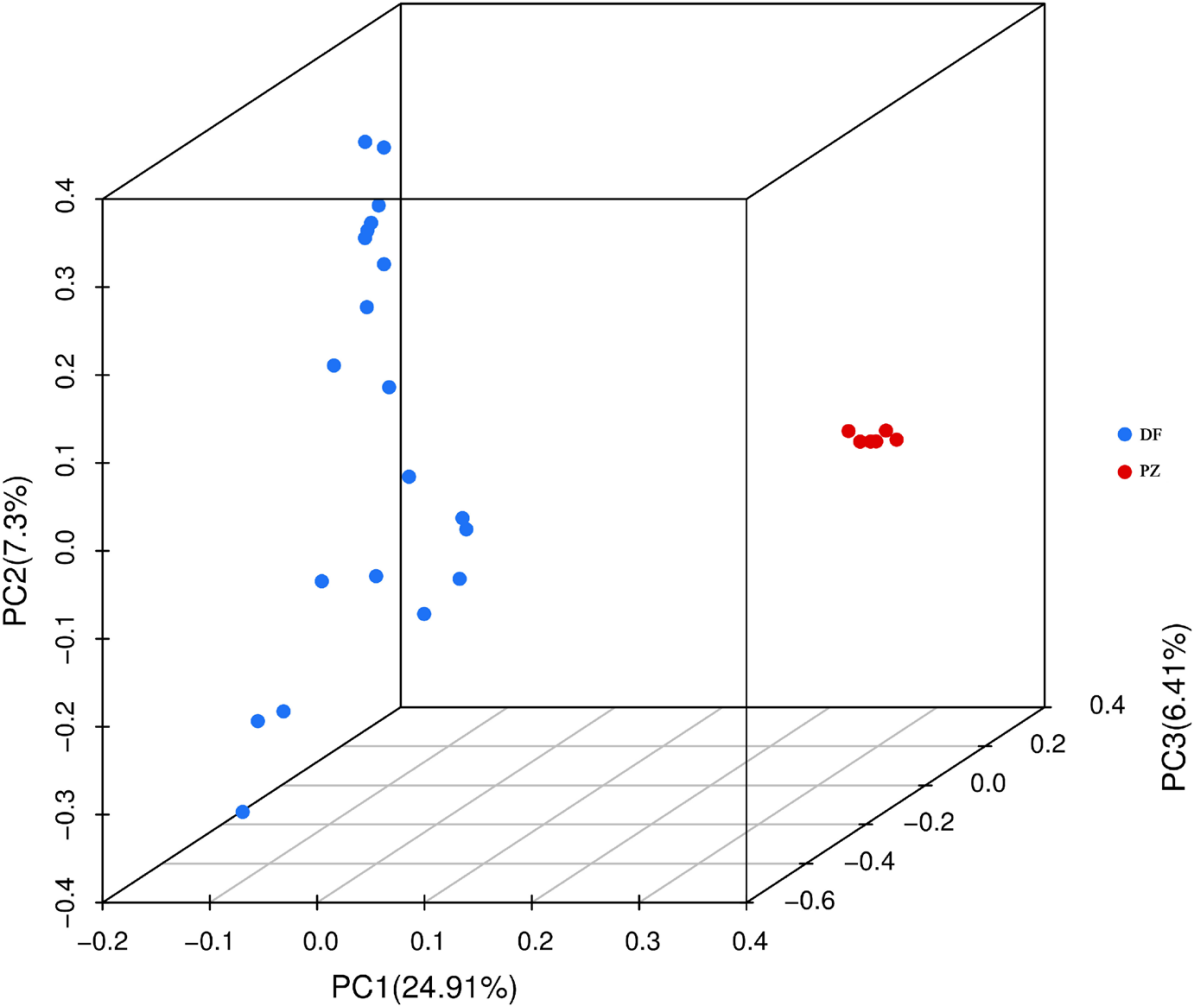
Principal component analysis of *R. bailiense* based on SNP.

To further analyze the genetic relationships of *R. bailiense*, this study employed GCTA software to perform Principal Component Analysis (PCA) using SNP data. As shown in Figure 7, the first principal component (PC1), the second principal component (PC2), and the third principal component (PC3) explain 24.91%, 7.30%, and 6.41% of the genetic variation, respectively. The DF population and the PZ population do not overlap, indicating significant genetic background differences between the two populations. This finding is consistent with the results of the genetic structure analysis.

### 3.8 Cluster Analysis

**FIGURE 8:**
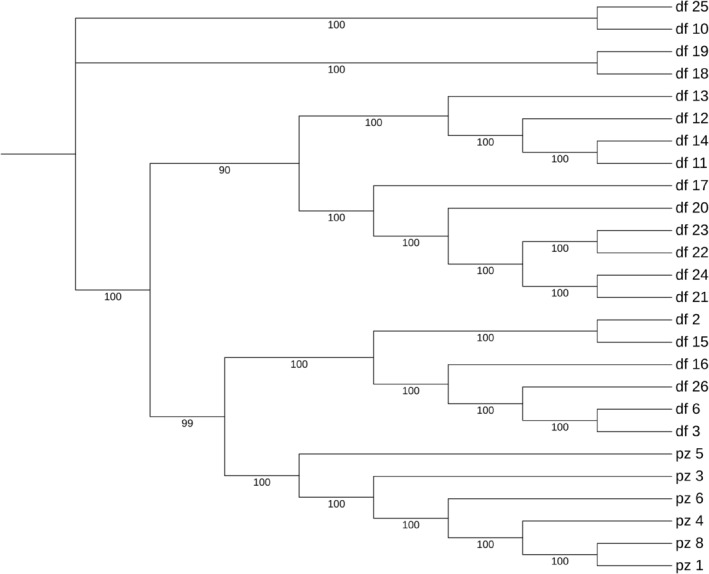
Phylogenetic tree of *R. bailiense* based on SNP.

Using the Maximum Likelihood algorithm in FastTree software, a phylogenetic tree was constructed (Figure 8), which is divided into five categories. The first category contains two samples, df10 and df25; the second category also contains two samples, df18 and df19; the third category includes 10 samples, namely df11, df12, df13, df14, df17, df20, df21, df22, df23, and df24; the fourth category comprises six samples, df2, df3, df6, df15, df16, and df26; the fifth category consists of six samples, pz1, pz3, pz4, pz5, pz6, and pz8. This classification aligns with the genetic structure observed when cross‐validation clustering at *K* = 5. The presence of extensive cross‐mixing among the DF population samples indicates a high level of genetic variation within this group, a finding consistent with the results of the genetic diversity analysis.

### 3.9 Population Demographic History Analysis

**FIGURE 9:**
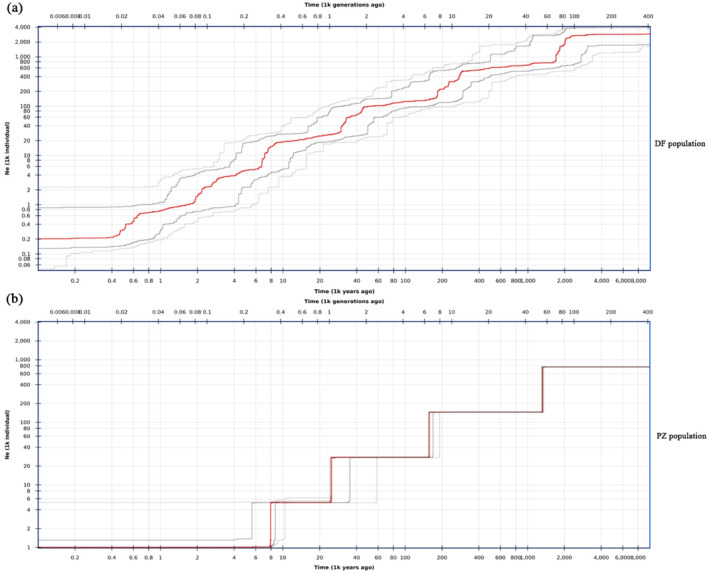
(a) Demographic history of DF population lineages by Stairway plot. (b) Demographic history of PZ population lineages by Stairway plot. Ne, Effective population size; 95% confidence interval is indicated by thin gray lines; the average is indicated by red lines.

Stairway Plot analysis based on the Site Frequency Spectrum (SFS) indicates (Figure 9) that the earliest historical timeline for the *R. bailiense* population can be traced back to 414 kaBP (kilo‐anniversaries Before Present). The initial effective population size (Ne) of the DF population was 2175 individuals. By 200 kaBP, during the Early Pleistocene (3 ~ 1 maBP) (maBP, million‐anniversaries Before Present), the DF population experienced six rapid expansions, reaching an Ne of approximately 275,000 individuals. Subsequently, the population size stabilized, maintaining stability through the Last Glacial Maximum (LGM) (26.5 ~ 19.0 kaBP) and into the Holocene (11.7 kaBP) until about 4000 years ago. The initial effective population size (Ne) of the PZ population was 925 individuals. By 60 kaBP, during the Middle Pleistocene (1 ~ 0.1 maBP) (maBP, million‐anniversaries Before Present), the PZ population underwent four rapid expansions, reaching an Ne of approximately 80,000 individuals. The population then remained stable until about 2000 years ago.

## 4 Discussion

### 4.1 Genetic Diversity of *R. Bailiense* Populations

The study of genetic diversity can reflect the potential for species to adapt to environmental changes, in addition to allowing an effective assessment of their current resources and modes of survival, especially for threatened species (He et al. 2024). The level of genetic diversity of a species is associated with multiple factors including its breeding system, ecological habits, geographical characteristics of its distribution area, climate changes, and evolutionary history (Hamrick and Godt 1996; Zhu et al. 2016). Research indicates that species with wide distributions generally have higher genetic diversity compared to rare and endangered species with narrow geographic distributions due to genetic drift and inbreeding (Hamrick and Godt 1996; Cole 2003). Compared to herbaceous plants, woody plants typically exhibit higher genetic diversity at the species level (Chung et al. 2020). For biallelic SNP markers, nucleotide diversity (*π*), observed heterozygosity (*H*_*o*_), and expected heterozygosity (*H*_*e*_) are overall indicators of population genetic diversity. The results of this study show that the genetic diversity (*π* = 0.2489; *H*_*o*_ = 0.2039; *H*_*e*_ = 0.2331) of the endangered woody species *R. bailiense* is relatively higher compared to herbaceous plants studied using ddRAD‐seq technology, such as *Dendrobium densiflorum* (*π* = 0.1056), *Geodorum densiflorum* (*π* = 0.0359), and *Viola uliginosa* (*π* = 0.0440) (Roy et al. 2017; Lee et al. 2020). Compared to other species within the same genus, Rhododendron species exhibit significant differences in genetic diversity, which may arise from varying levels of sexual reproduction, different population sizes, ages of species and populations, as well as differing pressures and selection (Li et al. 2019). For instance, species with few individuals in the population, such as *R. longipedicellatum* (*H*_*e*_ = 0.5610), *R. protistum* var. *giganteum* (*H*_*e*_ = 0.6020) (Wu et al. 2015), *R. hemsleyanum* (*π* = 0.2410; *H*_*o*_ = 0.1636; *H*_*e*_ = 0.2267) (Cao et al. 2022) and *R. sinofalconeri* (*π* = 0.2059; *H*_*o*_ = 0.1878; *H*_*e*_ = 0.1856) (Zhang et al. 2021), have higher genetic diversity and may retain genetic information from when their ancestors had a wide and continuous distribution. Conversely, *R. cyanocarpum* (*π* = 0.0702; *H*_*o*_ = 0.0442; *H*_*e*_ = 0.0675) (Liu et al. 2020), and *R. huadingense* (*π* = 0.00024) (Chen 2016) have lower genetic diversity, with habitat fragmentation leading to the gradual reduction in population size, causing inbreeding depression and loss of genetic diversity. *R. bailiense* genetic diversity is at a moderate level (*π* = 0.2489; *H*_*o*_ = 0.2039; *H*_*e*_ = 0.2331), indicating strong adaptability and significant potential for genetic improvement. Among these, the DF population exhibits higher genetic diversity than the overall level (*π* = 0.2489). The observed heterozygosity (*H*_*o*_) is less than the expected heterozygosity (*H*_*e*_), suggesting the presence of some degree of inbreeding or close breeding. The genetic effects of habitat fragmentation are gradually becoming apparent. According to the principles of conservation genetics, inbreeding is a trigger for genetic drift, which can reduce the species' survival and reproductive capabilities, increase the population's homozygosity, and lead to inbreeding depression. This could potentially result in a reduction in the population's genetic diversity. Therefore, when conserving the DF population, the phenomenon of close kin breeding should be considered. The genetic diversity of the PZ population is below the overall level (*π* = 0.2070), with observed heterozygosity (*H*_*o*_) greater than expected heterozygosity (*H*_*e*_), indicating a heterozygous selective advantage and better adaptability to the environment.

### 4.2 Genetic Differentiation of *R. Bailiense* Populations

The genetic differentiation coefficient (*F*_*ST*_) is an indicator used to measure the extent of allele frequency differentiation between populations (Excoffier and Lischer 2010). In this study, the average F_ST_ between two *R. bailiense* populations is 0.1907, indicating a high degree of differentiation (0.15 < *F*_*ST*_ < 0.25). Gene flow, primarily facilitated through the dispersal of pollen and seeds, is a major factor influencing genetic differentiation between populations. Previous research has found that Rhododendron seeds are dispersed by wind over distances of 30 to 80 m, whereas pollen transported by insects and birds typically travels between 3 and 10 km (Ng and Corlett 2000). However, the geographical distance between the two *R. bailiense* populations is approximately 16 km, making the exchange of pollen and seeds challenging. The long‐distance geographical isolation and specific habitats are likely to further hinder gene exchange between these highly isolated remnant populations in the future. The potential negative consequences include reduced gene flow between the remaining small populations and increased risk of genetic drift, which are major reasons for the high level of genetic differentiation observed in *R. bailiense*. The breeding system is often considered a critical factor affecting species genetic diversity. Both selfing and outcrossing breeding systems are recorded in Rhododendron, and previous studies indicate a lack of a typical self‐incompatibility system in Rhododendrons, suggesting a complex mating system (Ma, Wu, et al. 2015). Based on the 1‐IBS genetic distance matrix and the whole‐genome relationship matrix, the PZ population relatively smaller genetic distances, indicating some degree of selfing or close breeding. In contrast, the DF population larger genetic distances, with a clear advantage in outcrossing. Therefore, the breeding system may significantly impact the genetic differentiation of *R. bailiense*. AMOVA analysis revealed significant genetic variation both within and between populations, with greater variation between populations, consistent with findings from Ma, David, et al. (2015) using SNP markers to analyze genetic differentiation between populations of the endangered plant *Amygdalus ledebouriana*. This further supports the view that rare and endangered plants face restricted gene flow due to habitat fragmentation, inefficient seed dispersal mechanisms, and limited foraging areas for pollinators, with genetic variation primarily existing between populations (Fu et al. 2020). Therefore, conservation strategies should focus on protecting and maintaining genetic diversity across different populations while promoting moderate gene flow, considering ecological and geographical isolation factors, and implementing customized conservation measures. Such strategies help preserve the overall genetic health and adaptability of *R. bailiense*, enhancing its resilience to environmental changes. Cluster analysis, based on SNP data for population structure analysis and PCA, supports that *R. bailiense* populations are not a genetically homogeneous single group but consist of two genetically heterogeneous sub‐populations, with the DF and PZ populations originating from two different ancestors. The phylogenetic tree further divides the two sub‐populations into five categories, with the DF population exhibiting more genetic variation relative to the PZ population, consistent with the genetic diversity analysis results. Given the relatively low genetic differentiation between the two sub‐populations (*F*_*ST*_ = 0.1907), and considering the habitat characteristics and barriers such as valleys, human constructions, and farmlands between them, it is speculated that the sub‐populations may have arisen from habitat isolation and selective evolution driven by microenvironmental factors.

### 4.3 Population Demographic History of *R. Bailiense*

Historical dynamic studies indicate that the *R. bailiense* populations can be traced back to as early as 414 kaBP. The DF population experienced six population expansions during its evolutionary process. Its effective population size (*N*_*e*_) began to stabilize after the early Pleistocene, maintaining a stable state during the Last Glacial Maximum (26.5 ~ 19.0 kaBP) and throughout the Holocene (11.7 kaBP) until about 4000 years ago. The PZ population underwent four population expansions, with its effective population size (*N*_*e*_) stabilizing after the middle Pleistocene and remaining stable during the Last Glacial Maximum (26.5 ~ 19.0 kaBP) and throughout the Holocene (11.7 kaBP) until about 2000 years ago. This suggests that *R. bailiense* historically might have been a large population, which, due to the rapid uplift of the Yunnan‐Guizhou Plateau since the Pleistocene and the repeated fluctuations of the glacial and interglacial periods of the Quaternary, became fragmented into the two existing populations (Hewitt 2000; Zhou et al. 2017; Fan et al. 2022; Beckford et al. 2023). This aligns with Stebbins' proposed alternative evolutionary hypothesis (Zhuo et al. 1998; Liang 2020), where the high levels of genetic diversity might originate from an ancestral population. Therefore, we hypothesize that the current distribution pattern of the *R. bailiense* populations may be related to geological and climatic changes. Field surveys have found that the current effective population size of *R. bailiense* is much smaller than the recommended threshold to prevent loss of adaptability (*N*_*e*_ ≥ 100) and especially below the minimum suggested size to retain evolutionary potential (*N*_*e*_ ≥ 1000) (Frankham et al. 2014). Therefore, there is an urgent need to focus on and strengthen the protection of these remaining populations of *R. bailiense*.

### 4.4 Conservation Recommendations

Protecting the genetic diversity of species is crucial to prevent the loss of specific germplasm of that species, and it is of great importance for the maintenance of species diversity and long‐term survival. It is evident that the evolutionary potential, resistance to adverse environments, ecosystem resilience, and stability of a species are all contingent upon the magnitude of genetic diversity. Therefore, genetic variation information plays an important role in the formulation of plant conservation and management strategies. Although the *R. bailiense* population has high genetic diversity, its distribution range is narrow and the population size is small. To effectively maintain the genetic diversity of *R. bailiense* and achieve population size recovery, we propose the following suggestions: First, based on this study, the DF population has the richest genetic diversity and should be prioritized during germplasm collection and reintroduction. By designing seed collection strategies and building a seed germplasm resource bank, comprehensive collection of samples from the DF and PZ populations should be conducted, and plant tissue culture techniques should be used to preserve germplasm. Second, it is recommended to combine in situ conservation with ex‐situ conservation and reintroduction into the wild. Efforts should be made to establish a nature reserve in Panzhou City as soon as possible, and to strengthen the management of the Baili Rhododendron Scenic Area, strictly limiting the construction of tourist facilities to prevent human interference, and to manage nurturing effectively to protect its natural habitat. At the same time, by assessing the ecological factors of the current habitat of *R. bailiense*, suitable habitats for near‐natural ex‐situ conservation of seedlings should be found to expand the population distribution of *R. bailiense*, achieve population revitalization and natural return of artificial populations, ultimately avoiding the extinction of this species and enabling its effective protection and utilization. Third, artificial hybridization should be conducted among populations to rapidly increase heterozygosity. Further research on the asexual reproduction techniques of *R. bailiense* should be conducted, and rapid propagation should be achieved through tissue culture and cutting. After transplanting seeds collected from the field, seedlings should also be introduced back to the source location.

## 5 Conclusion

This study is the first to use ddRAD‐seq sequencing technology for Single Nucleotide Polymorphism (SNP) analysis, systematically analyzing the genetic background of *R. bailiense*. This species exhibits rich genetic diversity at both species and population levels, showing great potential for further selection and breeding. It displays a high level of genetic differentiation, with genetic variation primarily originating from between populations. This suggests that *R. bailiense* historically might have been a large population, which, due to geological historical events, became fragmented into the two existing populations. Throughout its evolutionary process, there have been multiple population expansions, with the DF population exhibiting more genetic variation compared to the PZ population. It is crucial to adopt a combined approach of ex‐situ and in situ conservation to expand the population size while maintaining high genetic diversity, which is vital for the recovery and utilization of *R. bailiense* populations.

## Author Contributions

**Jun Luo:** writing – original draft (equal). **Congjun Yuan:** writing – review and editing (equal). **Haodong Wang:** data curation (equal), investigation (equal). **Jianhua Zhang:** investigation (equal). **Jin Chen:** visualization (equal). **Shuang He:** formal analysis (equal). **Meng Chen:** investigation (equal). **Xiaoyong Dai:** resources (equal), writing – review and editing (equal). **Dali Luo:** investigation (equal).

## Conflicts of Interest

The authors declare no conflicts of interest.

## Data Availability

The raw sequence data reported in this paper have been deposited in the Genome Sequence Archive (Genomics, Proteomics & Bioinformatics 2021) in National Genomics Data Center (Nucleic Acids Res 2022), China National Center for Bioinformation/Beijing Institute of Genomics, Chinese Academy of Sciences (GSA: CRA016443) that are publicly accessible at https://ngdc.cncb.ac.cn/gsa.
